# Some Remarks on Internal Derangements of the Knee

**Published:** 1909-10-23

**Authors:** 


### Surgery.

### SOME REMARKS ON INTERNAL DERANGEMENTS OF THE KNEE.

As the result of any damage to the knee the internal semilunar cartilage is frequently displaced or torn. This injury may be regarded as, in itself, a slight affair; but there is hardly one which causes so much inconvenience. It not infrequently prevents the patient from following his occupation, and thus interferes with his wage-earning capacity.

It is produced in nearly all cases by a lateral movement, or forcible rotation of the tibia upon the femur, though the patient may be unaware of this in the excitement of the moment. Football is one of the most fruitful causes of the lesion. The sort of history that one commonly obtains from the patient is that in the course of the game he was thrown to the ground and his knee was "put out." He then discovered that he was unable to walk, and had to be carried home. Occasionally he will tell one that immediately after the accident his leg was bent up to a right angle or more, and he was unable to straighten it for himself, the intervention of the bystanders being necessary to extend the limb.

In a few hours the joint swells up owing to effusion into the synovial sac, whether there has been any injury to the cartilage or not. The immediate treatment consists of rest with the limb on a splint, and the application of an evaporating lotion to get rid of the effusion. At this stage it is impossible to say whether one is dealing with a simple synovitis or whether there is a more serious lesion causing the mischief. This point will have to be decided by the subsequent history. If the cartilage has been torn or displaced the knee will be put out again from time to time as soon as the patient gets about again. The slightest injury, such as stepping on an uneven piece of ground, is sufficient to produce this result; and on each such occasion there will be a return of the synovitis.

The chief thing that the patient complains of is a sense of insecurity in the affected joint. To put it in his own words, "the knee lets him down whenever he makes a sudden movement." In some cases he actually falls to the ground, but most often he means that the knee suddenly gives way and he is compelled to clutch at the nearest support to prevent himself from falling. Locking of the knee in the later stages is comparatively rare.

The structure that is usually torn or displaced is the internal semilunar cartilage. This is easy to explain. The internal cartilage is the larger of the two; and it is situated on the side of the joint where rotatory movement takes place. It will be remembered that the weight of the body is distributed through the external condyle and outer tuberosity; so that when a violent lateral twist occurs the internal condyle rotates on the inner tuberosity. Now the inner edge of the internal semilunar cartilage is firmly fixed to the capsule of the joint, and thus it is torn from the tibia to which it is attached by the coronary ligaments. But it is a mistake to suppose that it is only the internal cartilage which is damaged in this way, as the following case will show. A man, aged thirty-nine, slipped in unloading a ship and put out his right knee. He afterwards had symptoms which pointed definitely to a displaced semilunar cartilage. Exercises and massage were tried without benefit, and an operation was performed. The internal semilunar cartilage was found twisted upon itself, and the greater portion of it was removed. Convalescence was uneventful, but when he began to walk he still complained of pain in the knee, especially upon flexion, which was performed in a curiously spastic and jerky manner. It was supposed that he had some stiffness of the joint as the result of the operation, and he was made to do exercises for three months. But the condition still persisted. The joint was accordingly opened a second time and more thoroughly explored, and on this occasion it was discovered that the anterior extremity of the external cartilage was also loose. This was removed, and all his symptoms disappeared.

The main difficulty that one experiences in dealing with these cases is in deciding whether the cartilage is definitely damaged or not. All one has to rely on is the patient's history. Examination of the joint does not reveal any abnormality. It is quite exceptional to be able to feel the displaced cartilage extruded from the point. The most one can discover in an ordinary case is a slight clicking when the leg is flexed or extended at the knee.

The points in the history on which the greatest reliance should be placed are (1) the nature of the original accident, (2) the occurrence of repeated giving way of the knee, with subsequent synovitis, and (3) the history of locking. In cases in which the diagnosis can be made certain on these lines an operation should be advised, since palliative treatment is unlikely to relieve the symptoms. But should an element of doubt exist, massage and exercises should be tried for a month, with the object of strengthening the periarticular structures. Surgical instruments, designed to prevent any movement at the knee, except flexion and extension, are cumbrous and inefficient. If no improvement occurs in one month, an operation should be performed.

Many different incisions have been advocated for removing a semilunar cartilage. But, in the writer's opinion, it is best to turn up a flap, convex forwards, on the inner side of the knee, and open the joint horizontally at the level where the femur and tibia come in contact. It is better to do this than to make the skin incision coincide with that in the capsule. When the joint is opened there is rarely any difficulty in finding the cartilage. It may be simply displaced or bent upon itself or split horizontally. In any case, the whole of the distorted portion should be removed. It is not a good practice to attempt to straighten it out and fix it down to the tibia. All bleeding points should be carefully ligatured, and the openings in the synovium and capsule sewn up with catgut. Passive movement should be begun about the sixth day.

